# Use of Animal-Derived Products for Medicinal and Belief-Based Purposes in Urban Cities of Southwestern Nigeria: A One Health Perspective

**DOI:** 10.3390/ani16030502

**Published:** 2026-02-05

**Authors:** Samuel N. Akpan, Ralph Buij, Frank van Langevelde, Lian F. Thomas, Ayotunde E. Sijuwola, Olusola A. Ogunsanya, Pim van Hooft, Oluwatobi A. Adedokun, Abraham A. Adeyemo, Akeemat O. Ayinla, Dawn M. Zimmerman, Elizabeth A. J. Cook, Sherril P. Masudi, James M. Hassell, Christian T. Happi, Anise N. Happi

**Affiliations:** 1 Institute of Genomics & Global Health, Redeemers University, Ede PMB 230, Nigeria; sijuwolaa@run.edu.ng (A.E.S.); ogunsanyao@run.edu.ng (O.A.O.); adedokunolu@run.edu.ng (O.A.A.); abrahamrocketmail2@gmail.com (A.A.A.); akeematayinla@gmail.com (A.O.A.); happic@run.edu.ng (C.T.H.); happia@run.edu.ng (A.N.H.); 2 Wildlife Ecology & Conservation Group (WEC), Wageningen University & Research, Droevendalsesteeg 3a, 6708 WB Wageningen, The Netherlands; frank.vanlangevelde@wur.nl (F.v.L.); pim.vanhooft@wur.nl (P.v.H.); phyllis.masudi@wur.nl (S.P.M.); 3 International Livestock Research Institute (ILRI), Nairobi P.O. Box 30709-00100y, Kenya; lthomas8@ed.ac.uk (L.F.T.); e.cook@cgiar.org (E.A.J.C.); 4 Animal Ecology Group (AEG), Wageningen Environmental Research, P.O. Box 47, 6700 AA Wageningen, The Netherlands; ralph.buij@wur.nl; 5 Royal (Dick) School of Veterinary Studies, University of Edinburgh, Eastern Bush Campus, Midlothian EH25 9RG, UK; 6 Department of Entomology, Smithsonian Institution—National Museum of Natural History, 10th St. & Constitution Ave. NW, Washington, DC 20560, USA; zimmermand@si.edu; 7 Department of Epidemiology of Microbial Disease, Yale School of Public Health, New Haven, CT 06510, USA; hasselljm@si.edu; 8 Smithsonian Conservation Biology Institute, 3001 Connecticut Ave NW, Washington, DC 20013, USA; 9 Harvard T.H. Chan School of Public Health, 677 Huntington Ave, Boston, MA 02115, USA

**Keywords:** zootherapy, wildlife species, One Health, belief-based uses, zoonotic spillover, conservation, transboundary animal diseases

## Abstract

Zootherapy is a significant component of traditional medicine in many parts of the world. This study investigated the therapeutic and belief-based use of wildlife in urban settings in Southwest Nigeria, assessing its potential implications for One Health. We used semi-structured questionnaires, focus group discussions, and participant observations to gain insights into the practices, practitioners’ knowledge, motivations, and animal species used. Also, we searched the published literature and the International Union for Conservation of Nature Red List of Threatened Species for zoonotic pathogens and the conservation statuses of the reported animal species. Descriptive statistics and thematic analysis were used to analyze the data. Results revealed 53 practices involving 41 species, with occasional importation of animal parts to meet demand. Practitioners’ limited awareness of zoonotic risks, coupled with income generation and deeply rooted traditional beliefs, sustained these practices, further fueled by the dynamics of urban wild meat trade. The majority (95%) of species reported were reservoirs of zoonotic pathogens, with 44% in the vulnerable, near-threatened, endangered, or critically endangered class. The use of animal-derived products for medicinal and spiritual purposes may constitute a plausible pathway for potential zoonotic spillovers, transboundary animal diseases (TADs), decline in wildlife resources, and ecological imbalance. We recommend intensified monitoring, risk communication, awareness programs, and the adoption of sustainable alternatives to mitigate the negative impacts of these practices in Nigeria and beyond.

## Simple Summary

The use of animal-derived products for human healthcare and spiritual purposes, also called zootherapy, is practiced in many countries of the world, including Nigeria. We assessed the implications of therapeutic and belief-based use of wildlife products in southwestern Nigeria from a One Health lens. Income, traditional beliefs, and wild meat trade modulated these practices. Overall, 44% (18/41) of the animal species described by participants as abundant are classified as vulnerable or endangered under the IUCN Red List. Also, 95% (39/41) of reported species belonged to animal taxa documented as pathogen vectors and reservoirs, or hosts. Together, these results highlight the potential risks of zootherapy to public health, animal welfare, and environmental health.

## 1. Introduction

Traditional medicine remains a primary healthcare source for many communities in developing countries, where accessibility to modern healthcare is often limited [1]. Zootherapy, the use of animal-derived products for medicinal and spiritual purposes, plays a crucial role in indigenous healthcare systems across Africa, Asia, and Latin America [2,3]. It is deeply rooted in cultural and spiritual traditions [4,5]. In many parts of the world, animal-based treatments are used either independently or alongside plant-based treatments to address various physical and mental health conditions [6,7,8]. Zootherapy practices in Africa, Latin America, and Asia involve mammals, reptiles, birds, and insects [9,10]. Specific animal parts, such as bones, skins, organs, and secretions, are believed to have medicinal, spiritual, or symbolic properties [11]. For example, snake fat is used for its perceived anti-inflammatory effects, whereas crocodile skin is used in dermatological treatments [12,13]. In China and Brazil, pangolin scales and jaguar fat are similarly used for traditional healing [14,15].

However, the hunting and killing of wild animals for medicinal and belief-based purposes pose threats to wildlife conservation and public health. This could drive wildlife species to extinction. In addition, the use of wild animals in traditional medicine has been linked to emerging infectious diseases [16,17,18,19]. The processing and use of animal-derived products, often without proper hygiene or regulation, increases the risk of zoonotic spillover [10,14,20,21]. In China, for example, the illegal trade of wildlife for medicinal purposes has been implicated in the emergence of SARS-CoV-2 [22]. Likewise, in parts of Central Africa, it has been associated with the spillover of the Ebola virus [23]. The selection of animals for zootherapy is predominantly influenced by cultural beliefs, attributing healing properties to animal species believed to be associated with strength, endurance, or wisdom [15,24]. Preparation methods, such as drying, grinding, and mixing with herbs, are widely used [4].

The potency of zootherapeutic healthcare remains a subject of debate. While practitioners may believe in its efficacy, very few studies support these practices [25,26]. Although such studies are generally published in peer-reviewed complementary and alternative (CAM) medicine journals, they remain mostly unverified and may even be misleading [27]. Additionally, rigorous clinical trials and evaluations have largely not been conducted to assess various zootherapy potency claims [28]. Alternative medicine enthusiasts contend that scientific investigations cannot be applied to alternative medicine because the therapeutic approach is holistic and cannot be assessed via reductionistic science, the effects are too subtle to be measured, and the treatments must be customized for each patient, making them ineligible for clinical trials [29].

Southwest Nigeria, a densely populated multicultural region in sub-Saharan Africa, is a trade hub for wild meat and zootherapeutic practices [2,20]. A study [30] reported that “*Yoruba*” communities in southwestern Nigeria have practiced belief-based use of wildlife-derived products for many generations. Traditional healers, known locally as “*Babalawos*” (fathers of secrets), “*Iyalawos*” (mothers of secrets), or “*Ifá*” practitioners, play a central role in these traditions [31]. While studying the wild meat value chain in Lagos [20], we found that the medicinal and belief-based use of wildlife products in may be a risk factor driving wildlife exploitation, trade and public health risks. Hence, additional focus is needed on this subject as a distinct yet overlapping aspect of the wild meat trade in Lagos and other developing urban cities. Many studies conducted on this topic have been fragmented, focusing either on public health, anthropology, or on ecology and conservation [3,8,10,19,32,33,34].

Therefore, this study examines the implications of medicinal and belief-based wildlife uses through the holistic lens of One Health, which encompasses human, animal, and environmental health, drawing parallels to similar practices in other regions of the world. The findings from this study contribute to a broader global understanding of the intersection between traditional medicine, biodiversity conservation, and public health.

## 2. Materials and Methods

### 2.1. Data Collection

**Figure 1:**
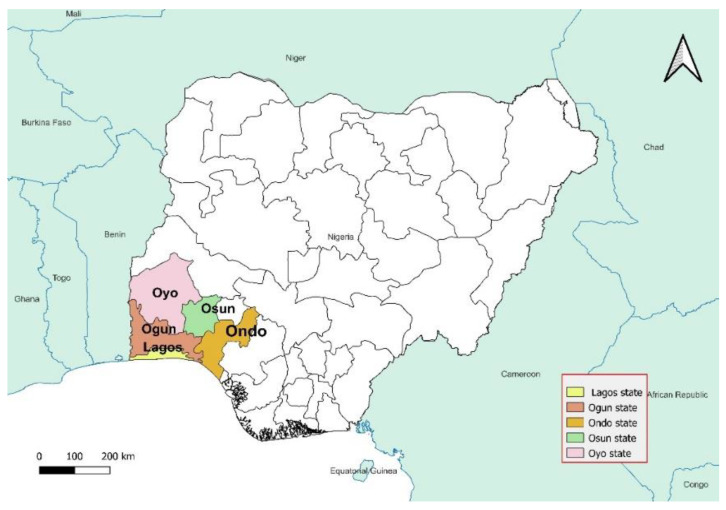
Map of Nigeria showing the study area.

A mixed-methods study approach was employed, incorporating questionnaires and focus group discussions (FGDs). The research team visited wildlife product sections in open markets and traditional healing homes across Lagos and four other states in southwestern Nigeria: Ogun, Oyo, Ondo, and Osun (Figure 1). The specific towns visited were Surulere, Ikeja, Epe, Abeokuta, Ijebu-ode, Ibadan, Oyo town, Akure, Ondo town, Osogbo, and Ilesha.

Using a local language interpreter, a purposive sampling approach was used to recruit study participants. Eligible participants included traditional healers, wildlife product traders/vendors, and individuals engaged in the use or sale of wildlife-derived products for medicinal or belief-based purposes. Although hunters were not directly recruited, hunting activities and supply chains were frequently described by participants during interviews and focus group discussions. Study sites were purposively selected using prior empirical evidence of high wildlife trade and zootherapeutic activity. Towns and markets identified as hotspots in a previous urban wild meat value chain mapping exercise were chosen based on high trade volumes, established networks, and overlap with medicinal and belief-based wildlife use [20]. This targeted selection aimed to engage active zootherapy practitioners and generate in-depth qualitative insights aligned with the study’s exploratory objectives.

Using a project information sheet (File S1), we described the project objectives and administered semi-structured questionnaires (File S2) to thirty-one (*n* = 31) study participants who gave consent (File S3). Additionally, using a guide (File S4), six (*n* = 6) FGDs were conducted with practitioners who declined to participate in questionnaire surveys but gave informed verbal consent to participate in focus group discussions (FGDs). The lead author documented verbal consent in a field log, noting participant codes, date, location, and activity type. Written consent was waived, as approved by the study’s ethics committee, due to the sensitive nature of the practices discussed and participants’ preference for non-written consent. All the participants gave verbal consent, as witnessed by the lead author. Additionally, the participants’ observations were recorded, and photos were taken of the wildlife body parts used for zootherapy, as seen by the researchers during the field data collection process.

### 2.2. Operational Definitions of Participant Categories

In this study, *traditional healers* refer to individuals who provide medicinal and belief-based services using wildlife-derived products, often with spiritual interpretations or rituals. *Wildlife product traders and vendors* were persons primarily engaged in the sales of wildlife body parts or products in market settings, regardless of whether they offered treatment services. Some participants held *overlapping roles*, combining healing, spiritual consultation, and product trading. ‘*Traditionalists*’ refers to participants who self-identified with African traditional religious belief systems and whose practices are rooted in indigenous spiritual and cultural frameworks. Recruited hunters did not practice as a profession, but were, however, knowledgeable about the practices, being learnt over time through family connections.

### 2.3. One Health Analytical Framework

This study used an integrative One Health framework to connect human behaviors, animal use, and related environmental and public health risks. Qualitative data from questionnaires and focus groups characterized human practices, beliefs, behaviors, and exposure pathways linked to zootherapeutic wildlife use. These findings were systematically linked to animal-level data, including species identity, body parts used, and conservation status, with references to the IUCN Red List. To assess health risks at the human–animal interface, species-specific zoonotic and veterinary disease hazards were identified through a targeted literature review. By synthesizing human practice data with species-level conservation and zoonotic risk information, the study enabled a unified assessment of public health, animal health, and biodiversity within the One Health framework.

### 2.4. Data Analysis

Species seen or photographed during field observation were phenotypically identified by experts on the research team, and the categorical data were analyzed via descriptive statistics (Table 1). We analyzed qualitative data from focus group discussions using thematic analysis, following Braun and Clarke’s approach [35]. Audio recordings were transcribed verbatim and checked for accuracy against field notes. We applied an inductive-deductive method, starting with data familiarization and systematic coding based on study objectives. Related codes were grouped into categories and refined into overarching themes through iterative review and comparison across transcripts. The research team reviewed and refined themes to ensure coherence and alignment with the dataset. Discrepancies were resolved through discussion, and final themes were selected for their relevance to the study objectives.

We also searched the International Union for Conservation of Nature (IUCN) red list of threatened species [36] for data on the conservation statuses of the animal species reported by the study respondents. To enhance our understanding of potential zoonotic risks, we conducted a literature search of two databases (Google Scholar and PubMed) for information on zoonotic pathogens that were associated with the reported species. We combined free-text keywords and controlled vocabulary (e.g., MeSH terms in PubMed) using Boolean operators as follows: (“zoonoses” [MeSH Term] OR “zoonotic diseases” OR “spillover”) AND (“pathogens” OR “viruses” OR “bacteria” OR “parasites”) AND (“wildlife products” OR “wildlife species” OR “wild animals”) AND (“zootherapy” OR “medicinal use” OR “ethnomedicine”).

## 3. Results

### 3.1. Questionnaires

#### 3.1.1. Socio-Demography

The results revealed an equal gender balance among the respondents (16 males, 15 females). Approximately half of the respondents (15/31) were between 51 and 65 years old, followed by 10 respondents (10/31) in the 66–80 age group. Most respondents (18/31) had 20–29 years of experience, followed by 30–39 years (5/31) and 0–9 years (4/31), respectively. Traditionalists constituted the largest group of practitioners (15/31), with primary school emerging as the most predominant level of education of the respondents (16/31), followed by informal education (10/31) and secondary school (5/31). This is shown in Table 1.

A total of 31 questionnaire responses and 6 FGDs were drawn from Lagos, Ogun, Oyo, Ondo, and Osun States. FGD participants’ sampling frame consisted of wildlife product traders (*n* = 2 per state), traditional healers (*n* = 3 per state), and wildlife hunters (*n* = 1 per state except for Ogun with two) (Table 2).

#### 3.1.2. Knowledge and Perceptions

“Family heritage” was the main route through which the participants acquired knowledge of zootherapy and belief-based use of wildlife products (Table 3).

Here, children were taught through the activities of their parents and grandparents and were expected to imbibe the practices or trade as part of their ancestral inheritance. Others acquired knowledge through their line of business (4/31), whereas some reported acquiring knowledge through dreams while they slept (3/31). Only two of the respondents said they learned from other practitioners. Furthermore, the majority of the respondents (20/31) believed that their practices did not cause any harm to or decline in wildlife populations (Table 3), indicating that participants viewed zootherapy as sustainable, citing the continued abundance of forests and wildlife species in Nigeria. However, three participants noted that although certain species (e.g., pangolins) had become scarce, they believed in the resilience of wildlife despite anthropogenic pressures. Additionally, all the FGD participants in this study said that the use of zootherapy for treatments was more effective than modern medicine, especially for difficult-to-treat diseases (chronic convulsions, epilepsy-like symptoms, persistent infertility, severe rheumatism, mental or spiritual afflictions), which they believed were caused by demons.

### 3.2. Focus Group Discussions

Thematic analysis of the FGDs revealed the 6 themes: (i) species and practices, (ii) hygiene and safety, (iii) urban preference, (iv) importation (v) wild meat, and (vi) traditional beliefs. Table 4 presents the overarching themes, codes (sub-themes), and their descriptions.

#### 3.2.1. Species and Practices

The study participants reported a total of fifty-three (53) practices, cutting across forty-one (41) wildlife species. Some participants, however, declined to comment on the use of four species and three products (denoted by *), citing the need to keep the information discreet. The reported species, their products, associated zoonotic pathogens, and their conservation statuses are shown in Table 5.

These were (i) forest/jungle species: red-bellied monkeys, mona monkeys, patas monkeys, blue duikers, sitatungas, red-fronted gazelles, black-bellied pangolins, tree hyraxes, white-bellied pangolins, forest cobra, Nigerian shrews, giant-pouched rats, house rats, common warthogs, red river hogs, civets, and Ahanta spurfowls; (ii) savanna/grassland species: African hares and brush-tailed porcupines; (iii) wetland species: dwarf crocodiles, slender-snouted crocodiles, and mud turtles; (iv) desert species: monitor lizards; and (v) general species: Ahanta spurfowls, rock pythons, ball pythons, puff adders, spotted linsangs, Gaboon vipers, chameleons, bats, house geckos, owlets, vultures, and tortoises. In terms of specific practices, our study revealed that the whole heads of Nigerian shrews and Gambian giant-pouched rats are believed to have the ability to cure demon-related illnesses and headaches. Hare and gazelle limbs are believed to enhance athletic performance, whereas crocodile bones are thought to counteract poisons. Vulture heads are used to treat epilepsy, porcupine spikes are believed to offer spiritual protection, and tortoises are used for spiritual healing, while some species’ applications remain undisclosed (Table 5).

The participants also reported that monkey bones and ligaments were used to treat arm injuries in football goalkeepers, whereas python spinal vertebrae, intestines, and fat aid in backache, arthritis, and antivenom production. The bones of Ahanta spurfowl (popularly referred to as “bush fowl” by the locals) were used to strengthen children’s bones, whereas the Gaboon viper vertebrae were used for the treatment of diseases believed to be of spiritual origin. Bile, tongue, and fat derived from forest cobras contribute to antivenom and skin remedies. Pangolin scales were used to correct fetal malposition, and bush-pig snouts were used to treat fever in infants.

#### 3.2.2. Hygiene and Safety

With respect to safety concerns related to the processing and preparation of wildlife-derived products for traditional medicines, a participant stated:

“*Wild animals are from the wild, which is pure, so most of them don’t have any germs. However, we usually heat the animal parts on fire, to kill anything [germs] that may be there*”.

Another FGD participant stated:

“*We can use the same product for different people. For example, one can use the same monkey hands [forelimb] to cure stealing addiction [kleptomania] for up to 20–30 persons. There is no issue of passing [transmitting] any germs to people by contact with the same animal part…..it is not possible*”.

#### 3.2.3. Product Importation

With respect to the sourcing of the products, two FGD participants reported that, owing to the unavailability of certain animal-derived products, they sourced supplies from nearby countries: Cameroon and the Republic of Benin. However, no participant in this study reported exporting products.

Excerpts:

“*…several times the animal part I need is not available in our area……an animal like a leopard, for example. Sometimes the animal may be in our area, but the hunters are not lucky enough to see it. In that case, I talk to some connections in that place after you cross from Lagos….Benin. When I send money, they send it [the products] for [to] me*”

—FGD participant

“*Apart from also treating people, I also supply the products to some of our people, especially the elderly ones who cannot move around as before. When we don’t have some things here, I get the ones some traders bring from Cameroon and the Benin Republic.*”

—FGD participant

#### 3.2.4. Urban Preference

The FGD participants’ responses indicated that the practices did not differ between rural and urban areas. However, there was a growing interest of zootherapists in practicing in urban areas due to income considerations (i.e., the financial strength of clients in urban areas). This is seen in the following excerpts:

“*Some of us live in the rural villages but come here to treat people, because here there are many clients, they have the money, and they usually pay better for our services…hahahaha [laughs]*”

—FGD participant

“*Look, many big men [wealthy persons] were coming to my town to meet me, but sometimes they complain, because it is very far and the road is bad. Because of that, with the help of my eldest daughter, I moved to this part of Lagos. Since I am here, it is very easy for clients to visit me and refer other people who need me*”

—FGD participant

“*…being in the city is good for us. Apart from making more money, the bushmeat market in the city is bigger, and one can get the attention of many hunters and get the needed raw materials.*”

—FGD participant.

#### 3.2.5. Wild Meat as a Modulator

FGD participants reported that they or other persons had consumed meat from the same animals from which they derived products for medicinal or belief-based uses. The participants who were traditional hunters stated that the demand for wild meats and wildlife products believed to have medicinal properties often stimulated their hunting expeditions and that one cannot be separated from the other, as shown in the following excerpts:

“*The same trader selling bushmeat is the one who will sell crocodile head to you, if you need. It is the same thing. Although some of us only deal with wildlife parts used for medicine and other spiritual work, many deal with the two [both] because it is [they are] the same thing.*”

—FGD participant.

“*When we hunt a snake for their delicious meat, the first thing we do is to cut off the head and sell it to any traditional medicine dealer, or we use it by ourselves… You cannot waste it, because apart from the meat, every part of the animal is useful for other things. Then, we sell the meat to those who want to eat.*”

—FGD participant.

“*Many hunters don’t agree to enter the bush to hunt because of one tiny animal part that we need. They ask us to wait until they get a demand for bushmeat, then they go. It is from the bushmeat that they bring to us any animal parts that we need, like monkey skull*”

—FGD participant.

#### 3.2.6. Traditional Beliefs

In response to questions about the potential risks of zoonotic disease through human–wildlife contact, a participant stated:

“*Nature does not bless and curse. That is why wild animals do not pass any disease to us or anyone. As far as we are concerned, wild animals are clean [pure], and the forest is clean [pure].*”

On the efficacy of their practices, another participant stated:

“*….it is part of our tradition. Every community usually has at least one of us to solve problems….or they go to where they can find us. That is the only way to handle certain [medical, spiritual] cases among our people…..and it is very effective.*”

### 3.3. Literature Search Results

#### 3.3.1. Documented Zoonotic Pathogens

Our literature search results revealed multiple zoonotic pathogens associated with 38 reported species. These were viruses (e.g., Lassa fever virus, Rift valley fever virus, Influenza A, Ebola, Marburg, Nipah, SARS-CoV-2, MERS-CoV, Rabies, and Hantaviruses), bacteria (e.g., *Salmonella* spp., *Leptospira* spp., *Klebsiella* spp., *Campylobacter* spp., *Yersinia* spp., and *Chlamydia psitacci*), and parasites (e.g., *Toxoplasma gondii*, *Trypanosoma cruzi*, and *Hymenolepis* spp.) (Table 5). No data was found for 3 reported species (the red-fronted gazelle, the Ahanta spurfowl, and the West African linsang). While the documented presence of zoonotic pathogens does not imply direct transmission through zootherapeutic use, it does, however, indicate that potential exposure risks exist with handling, processing, and reuse of wildlife-derived materials.

#### 3.3.2. IUCN Conservation Statuses

The search results showed that three (3) of the reported species were critically endangered. These were the slender-snouted crocodile, the hinge-back tortoise, and the hooded vulture. Others were classified as endangered (3), near threatened (6), vulnerable (6), and of least concern (23) [36].

## 4. Discussion

### 4.1. Limited Awareness of Zoonotic and Public Health Risks

Education equips individuals with the knowledge, attitudes, and skills to recognize risks and prevent harm to themselves, their environment, and the public [70]. Hence, the low level of education observed among participants in this study suggests that practitioners may have engaged in risky practices, at least partly due to insufficient knowledge and awareness, as alluded to by Masudi et al. [71]. Our results also highlighted a knowledge system not aligned with Western biomedical paradigms, reflecting a global challenge where traditional medicine practitioners often lack formal education on zoonoses. As seen in Table 4, bats, primates, and rodents, which were all reported by the participants in this study) are globally documented reservoirs of zoonotic pathogens [10].

Therefore, the general belief in the purity of wildlife may predispose practitioners and their human patients to zoonotic infections. These perceptions reflect non-incidental and coherent belief systems that shaped daily practices. The risk perception gap is evident in a participant’s statement that ‘nature does not bless and curse,’ which reveals a strong belief in the inherent purity of wildlife and the natural environment. Such views support the rejection of wildlife-human disease transmission risks and help explain why repeated handling and reuse of animal-derived products are seen as safe, despite documented zoonotic hazards.

Furthermore, the predominance of practitioners older than 50 years (25/31) in this study is consistent with previous studies, which report that zootherapy and belief-based use of wildlife are practiced primarily among older individuals [7,72]. We posit that the low level of involvement among younger persons may be due to increased knowledge of disease concerns related to zootherapy or a gap in intergenerational knowledge transfer. This gap is usually attributed to globalization and urbanization, which influence younger individuals to adopt modern lifestyles, distancing them from traditional practices [73].

A triangulation of participants’ years of experience, age range, and mode of knowledge acquisition results suggests that zootherapy requires extensive time to master, often through apprenticeships and intergenerational knowledge transfer. Consistent with this finding, previous studies have stated that traditional medicine serves as a repository of ancestral knowledge, passed down verbally through generations [1,74,75]. However, reliance on verbal transmission through family heritage, rather than formal documentation or scientific validation, often contributes to the dissemination of misinformation and unsafe practices [2]. A study conducted in Uganda reported that the predominance of family heritage as the primary mode of knowledge acquisition limited exposure to modern medical and conservation principles [76]. Some respondents (3/31, 9.7%) in our study reported acquiring zootherapy knowledge of their practice through dreams, further emphasizing the mystical dimensions of these practices. This phenomenon is particularly evident in shamanistic cultures, where dreams are considered conduits for spiritual guidance and the transmission of healing knowledge [77]. For example, in some indigenous communities, individuals with shamanistic abilities may experience dreams that provide insights into medicinal practices, such as the use of animal parts for healing purposes [78]. While such practices may be culturally significant, they lack scientific validation [33], potentially leading to misinformation and unsafe health applications.

### 4.2. Income, Wild Meat, and Traditional Beliefs as Drivers

Excerpts from FGD responses showed that practitioners’ preference for urban centers was mainly due to income, fuelled by the perceived societal status of individuals in urban areas, and the need to improve clients’ ease of access to their services. Consistent with this finding, Coals et al. [33] report that wildlife products and services are traded and utilized across geographic and socioeconomic gradients, including urban cities, due to their integration into the global economy. This suggests that these practices may become increasingly prevalent in urban areas due to rural–urban migration driven by socio-economic factors. Also, our study findings pointed to an intricate web linking zootherapy with the wild meat trade and consumption. This aligns with findings from other studies that reported that zootherapy and the wild meat trade were integrated and interdependent [79,80], as the same harvested animals served both purposes. While our study results did not clearly establish the extent to which the demand for animal products influenced wild meat supply, our findings suggest that wild meat demand played an important role by modulating the frequency and availability of animal parts for zootherapy. Strong cultural beliefs related to zootherapeutic efficacy further drove the practices [2]. Traditional medicine is often intertwined with religious and mystical elements, necessitating culturally sensitive public health interventions that acknowledge these beliefs while promoting safer and more sustainable practices [81,82]. Religious affiliations play a crucial role in shaping the perception and practice of zootherapy [7]. Hence, the involvement of practitioners with different religious beliefs suggests that there may be variations in the application of zootherapy based on religious doctrines [83]. However, the high number of traditionalists in this study suggests a strong connection between these practices and the African traditional religious belief systems. This corroborates the findings of Akpan et al. [20], who reported that wildlife-associated practices in the study area were markedly influenced by the traditional beliefs of the “*Yorubas*”, the predominant tribe in southwestern Nigeria.

### 4.3. Animal Welfare and Conservation

Although the majority of participants (20/31) in this study expressed the belief that their practices caused no harm to the wildlife population, current conservation realities suggest otherwise. Nigeria faces challenges with the overexploitation of wildlife for medicinal use [34]. Trade in vulture parts for traditional healing has driven several vulture species towards extinction, which threatens their crucial ecological role in carcass disposal and disease prevention [30]. Similarly, chameleon overharvesting for traditional medicine and rituals has resulted in population reduction, putting the species at risk of extinction in particular locations [34]. The poor knowledge expressed by participants and the lack of scientific evidence to support the potency claims of their practices underscore the urgent need for innovative approaches to prevent people from further indulgence in these practices to mitigate the progressive overexploitation of already declining wildlife resources in Nigeria. The reliance on wildlife products for these practices may raise concerns regarding the overharvesting of wildlife populations. The majority (23/41) of animal species reported in this study were classified under the least concern category [36]. Considering the progressive overexploitation of wildlife, Akpan et al. [20] argue that the classification of many West African species is outdated and may not reflect their true statuses. Others, such as the hinge-back tortoise, slender-snouted crocodile, and hooded vulture, were critically endangered [36], necessitating stronger conservation measures across the study areas. Previous studies have demonstrated that unregulated wildlife use can drive species to extinction, as observed in the case of pangolins, which are now critically endangered because they are poached for traditional medicine and international trafficking [84]. Without immediate conservation interventions, the depletion of these wild animal species could have cascading ecological consequences, including the loss of critical ecosystem services, such as the natural pest control services provided by pangolins and vultures [85,86].

### 4.4. Zoonotic and Veterinary Disease Transmission Risks

Participants stated that they heated the animal-derived parts before use. While the heating or burning process of wildlife products (as seen in the excerpt) may inhibit certain heat-labile zoonotic pathogens that may be present, heat treatment is not always effective. Although many foodborne pathogens (e.g., *Salmonella* spp., *E. coli*) can be killed when exposed to high temperatures of 65 °C and above [87], some pathogens cannot be destroyed by high temperatures. For example, the spores of *Clostridium botulinum* and *Bacillus anthracis* (causative agents of botulism and anthrax) can withstand intense heat for extensive periods of time [88,89]. Furthermore, given the unstandardized and unmonitored state of the practices, the heating temperature (and duration) deployed by practitioners for the disinfection of animal products is unknown. Proper heating does not eliminate risks from prions, heat-stable toxins, or antimicrobial residues in animal tissues. This underscores the limitations of relying only on thermal processing for ensuring product safety. Also worthy of note is that the association of zoonotic pathogens with wildlife species does not imply their direct transmission through zootherapeutic use, but an indicator of potential zoonotic exposure risks.

Recent disease outbreaks highlight the serious health and socioeconomic impacts of zoonotic spillover events [19,90,91]. In zootherapeutic practices, the lack of standardized handling and processing may increase the risk of disease exposure for traditional healers and clients [92,93]. Limited regulatory oversight of wildlife-based medicine may further increase the risk that contaminated animal products may be used, thereby increasing zoonotic transmission risk [23,94]. Studies have reported the use of animal waste, such as urine and faeces, in human treatment [95], which may facilitate the spread of various zoonotic pathogens. Antimicrobial-resistant bacteria, including *Bacillus* spp. and *Staphylococcus* spp., have been found in cow urine used for zootherapy [96], raising concerns about the transfer of antimicrobial resistance from animal-derived materials to humans.

In addition to local zoonotic risks, the unregulated cross-border movement of wildlife-derived products identified in this study could expand regional health impacts. In Nigeria, porous international borders and informal wildlife trade networks allow animal tissues to cross national boundaries with little veterinary oversight and this may facilitate the risk of pathogen introduction and spread to wildlife, livestock, and food supplies. This movement may raise the risk of transboundary animal diseases (TADs), such as foot-and-mouth disease, African swine fever, anthrax, peste des petits ruminants, lumpy skin disease, and highly pathogenic avian influenza [97]. These diseases threaten animal health systems, livelihoods, and cause significant economic losses [97]. As a result, unmonitored cross-border sourcing of wildlife products complicates disease control and highlights the need to integrate wildlife trade surveillance into broader TAD prevention and One Health strategies.

### 4.5. Synergistic One Health Feedback Loops Linking Cultural Practices, Wildlife Exploitation, and Zoonotic Risks

**Figure 2:**
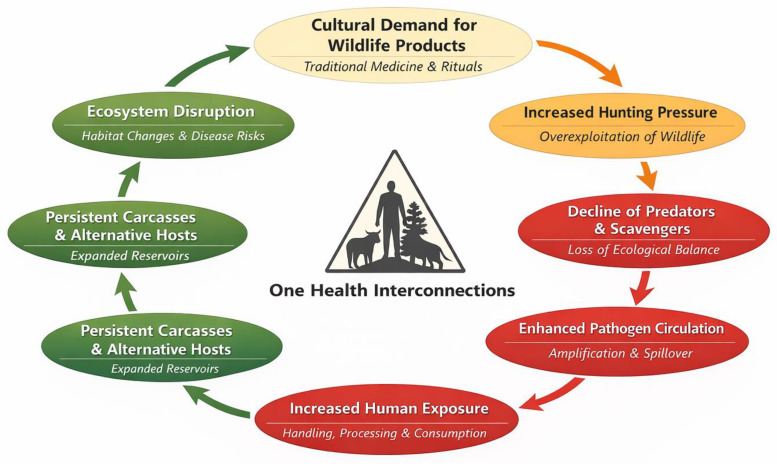
The conceptual One Health framework illustrates how reported zootherapeutic practices and wildlife trade pathways correspond to human exposure routes, conservation concerns (IUCN Red List status), and system-level drivers including urbanization, market accessibility, and informal importation. The diagram emphasizes reinforcing feedback loops among cultural demand, hunting pressure, wildlife population decline, ecological disruption, pathogen amplification, and increased human exposure risk, thereby demonstrating the interconnectedness of human, animal, and environmental health outcomes.

Looking at this issue through a One Health perspective, cultural demand for wildlife-based medicinal products creates feedback loops that go beyond the use of specific species. As shown in Figure 2, increased hunting pressure reduces key scavengers and predators, altering how carcasses are removed and the makeup of animal communities. These shifts in ecosystems, both over time and across different areas, can facilitate the spread and circulation of pathogens, as seen in the range of zoonotic agents listed in Table 5. This can increase the risk of human exposure to these pathogens through repeated handling, processing, or consumption of wildlife products. These feedback loops show how closely human, animal, and ecosystem health are linked, and why broad prevention and policy measures are needed.

Given the relatively small sample size, the quantitative findings of this study should be interpreted as descriptive indicators rather than statistically representative estimates. The percentage values contextualize observed patterns and support qualitative interpretations, rather than reflecting population-level prevalence. The principal strength of this research lies in its in-depth qualitative insights, thematic analysis, and the integration of species-specific zoonotic and conservation evidence within a One Health framework. Consequently, this study should be considered an exploratory investigation intended to generate hypotheses and inform future large-scale, quantitatively robust research.

## 5. Conclusions

Our study demonstrates that wildlife exploitation for medicinal and belief-based purposes persists, driven by deeply rooted cultural and spiritual worldviews. Participants described belief systems where illnesses are often seen as spiritual or demonic in origin, requiring the use of wildlife-derived products in conjunction with ritual practices. These findings indicate the presence of a distinct epistemological framework, rather than a lack of knowledge, which differs from Western biomedical paradigms.

From a One Health perspective, these practices may pose public health, conservation, and ecological risks, as indicated by literature on zoonotic pathogens, wildlife population decline, and ecosystem disruption. However, this study did not directly assess disease transmission, health outcomes, or therapeutic efficacy, so the effectiveness and safety of wildlife-derived products remain unverified. Hence, the presented implications of these practices should be viewed as potential risks based on documented practices and published evidence, not as proven causal effects.

Given the strong link between zootherapeutic practices and the urban wild meat trade, integrated surveillance at bushmeat markets and monitoring wildlife volumes, species composition, and health hazards offer a practical One Health entry point. Risk communication may be more effective if it targets high-risk taxa identified in this study, such as primates, bats, rodents, and pangolins, rather than using generalized wildlife messaging. Additionally, since practitioners already use heating as a perceived safety measure, co-developing standardized and culturally acceptable processing guidelines with stakeholders could help reduce exposure risks while respecting traditional beliefs.

Promoting scientifically validated, sustainable alternatives, such as plant-based or synthetic substitutes where appropriate, may help reduce reliance on wildlife-derived products if these options are effective and environmentally sound [98,99]. Overall, these findings highlight the need for culturally sensitive, evidence-based One Health approaches that engage practitioners, address urban trade dynamics, and balance public health, biodiversity conservation, and socio-cultural factors.

## 6. Limitation

This study’s main limitation was the small sample size of questionnaire responses and FGDs, due to the low number of practitioners who agreed to participate in the study, as only a few individuals were said to possess the knowledge of zootherapy. Some identified practitioners and individuals who had received zootherapy treatments declined participation, citing the need for secrecy in their business or fear of stigmatization. We acknowledge that small sample sizes result in limited transferability/generalizability, potential selection bias, and social desirability bias, which may impact the accuracy of research conclusions. However, based on the available sample size, we strived for a good representation of the practitioners’ practices, knowledge, and perceptions in the study area. Additionally, our reliance on self-reported data may have introduced potential biases, as respondents may have underreported or exaggerated certain aspects of their practices owing to the cultural sensitivity of this subject and livelihood concerns.

## Figures and Tables

**Table 1:** Socio-demography of the questionnaire respondents.

Category	Religion	Frequency (%)
Gender	Male	16 (51.6)
	Female	15 (48.4)
Age range	21–35	2 (6. 5)
	36–50	4 (12.9)
	51–65	15 (48.4)
	66–80	10 (32.3)
Religion	Traditionalist	15 (48.4)
	Muslim	9 (29.0)
	Christian	5 (16.1)
	Other	2 (6.5.)
Experience	0–9	4 (12.9)
	10–19	3 (9.7)
	20–29	18 (58.1)
	30–39	5 (16.1)
	40–49	1 (3.2)
Education	Primary school	16 (51.6)
	Secondary school	5 (16.1)
	Tertiary education	0 (0)
	Informal education	10 (32.3)
	None	0 (0)

**Table 2:** Distribution of study participants.

State	Questionnaire Respondents (*n* = 31)	FGDs (*n* = 6)	FGD Participants (*n* = 30)	FGD Participants Sampling Frame		
				Wildlife Product Traders (*n* = 10)	Traditional Healers (*n* = 15)	Wildlife Hunters (*n* = 6)
Lagos	6	1	7	2	3	1
Ogun	7	1	6	2	3	2
Oyo	6	1	5	2	3	1
Ondo	6	1	6	2	3	1
Osun	6	2	6	2	3	1

FGD = Focus Group Discussion.

**Table 3:** Participant responses on knowledge acquisition, conservation, and public health risks.

Questions	Responses	Frequency (%)
How did you acquire the knowledge of your profession?	Through family heritage	20 (64.5)
	Through the line of business	4 (12.9)
	Other means (i.e., dreams)	3 (9.7)
	Learnt from another practitioner	2 (6.5)
	Prefer not to say	2 (6.5)
Do you think that these practices can cause harm to the wildlife population?	Yes	11 (35.5)
	No	20 (64.5)
Do you think that these practices have any public health risks?	Yes	5 (16.1)
	No	26 (83.9)
Do you think that these practices can have an impact on the environment?	Yes	0 (0)
	No	27 (87.1)
	I don’t know	4 (13.0)

**Table 4:** Code book showing themes, subthemes, and their descriptions.

Themes	Codes (Subthemes)	Description
Species and Practices	Species	For comments on the wildlife species used by study participants
	Uses	For comments describing practices and the utilization of wildlife-derived products
Hygiene and Safety	Products hygiene	Used to capture comments on hygiene measures taken by participants
	Products safety	Used to capture comments on the safety of wildlife-derived products
Importation	Products scarcity	For comments on the scarcity of local species or their products
	Products sourcing	Captures comments on the sourcing of products internationally
Urban preference	Income	Captures comments on the financial motivations of participants
	Accessibility	Captures comments on participants’ ease of access to clients, and clients’ ease of access to their services
Wild meat	Products demand	Captures comments on the local demand for products
	Wild meat trade	Captures comments on wild meat hunting and supply
Traditional beliefs	Zoonoses	Captures comments on participants’ beliefs and perceptions regarding wildlife-human disease transmission risks
	Culture	Captures comments on cultural beliefs and norms

**Table 5:** Wildlife species, conservation statuses, derived products, uses, and associated zoonotic risks.

Common Name	Scientific Name	Conservation Status	Body Part	Uses	Documented Pathogens
Red-bellied monkey	*Cercopithecus erythrogaster*	Endangered	Skull Forelimbs	To ward off spiritual attacks To enhance the agility of football goalkeepers	Viruses: Simian foamy virus, Simian retrovirus D, Mpox virus, Rotavirus, Herpesviruses Dengue virus, Yellow fever, Marburg, Mpox, Ebola, Simian haemorrhagic fever virus; Parasites: *Schistosoma* spp. [37,38]
Mona monkey	*Cercopithecus mona*	Near threatened	Forelimbs	Treatment of arm injuries	
Patas monkey	*Erythrocebus patas*	Near threatened	Hands Head	To cure kleptomania; For good luck in business and career	
African rock python	*Python sebae*	Near threatened	Spinal vertebra Intestines Fats	For treatment of back pain; or snake anti-venom, treatment of arthritis	Bacteria: *Salmonella* spp., *Lysobacter pythonis*; Parasites: *Armillifer* spp. [39,40,41]
Ball python	*Python regius*	Near threatened			
Bushbuck	*Tragephalus scriptus*	Least concern	Bones	For fixing bone defects and fractures To enhance speed in one’s pursuits	Bacteria: *Leptospira* spp., *Coxiella burnetii* (Q fever) [42,43]
House gecko	*Hemidactylus* spp.	Least concern	*	*	Bacteria: *Salmonella* spp. [44,45]
African civet	*Civettictis civetta*	Least concern	Anus	To treat convulsions; to add a fragrance to one’s life	Bacteria: *Leptospira* spp. [42]
African palm civet	*Nandinia binotata*	Least concern			
Ahanta spurfowl	*Pternistis ahantensis*	Least concern	Bones	For strengthening children’s bones	Unknown
Gaboon viper	*Bitis gabonica*	Vulnerable	Spinal vertebrae	For spiritual diseases, eye infections, pain, and convulsions	Bacteria: *Mycobacterium* spp., *Salmonella* spp., *Chlamydia* spp., *Leptospira* spp., *Klebsiella* spp., *Campylobacter* spp., and *Yersinia* spp. [46]
West African carpet viper	*Echis ocellatus*	Least concern	Fangs	For snake anti-venom	
Puff adder	*Bitis arietans*	Least concern	Fats, head, and teeth	For rheumatism, and snake anti-venom	
Forest cobra	*Naja melanoleuca*	Least concern	Tongue, bile, and oil	For skin ailments, arthritis, and snake anti-venom	
Common duiker	*Sylvicapra grimmia*	Least concern	Bones	For the healing of bone fractures, to enhance athletes’ speed.	Bacteria: *Leptospira* spp. [42]
Black duiker	*Cephalophus niger*	Least concern			
African wildcat	*Felis lybica*	Least concern	Whiskers	*	Parasites: *Toxoplasma gondii* [47]
Pearl-spotted owlet	*Glaucidium perlatum*	Least concern	*	For curing issues caused by enemies	Bacteria: *Chlamydia psittaci* [48]
Black-bellied pangolin	*Phataginus tricuspis*	Vulnerable	Tail Whole	For the correction of malpositioned fetuses in pregnant women	Viruses: SARS-CoV-2, Human parainfluenza virus 3 (HPIV3), Human respiratory syncytial virus (HRSV) [49,50]
White-bellied pangolin	*Phataginus tetradactyla*	Endangered			
West African linsang	*Poiana leightoni*	Vulnerable	Whiskers	*	Unknown
Common warthog	*Phacochoerus africanus*	Least concern	Snout Fats	Treatment of fevers in children and rheumatoid arthritis	Viruses: Rift Valley fever virus, Influenza A virus; Bacteria: *Mycobacterium bovis*, *Brucella* spp., *Leptospira* spp. [51]
Red river hog	*Potamochoerus porcus*	Least concern			Bacteria: *Shigella* spp., *Salmonella* spp., *Campylobacter* spp. [52]
Nigerian shrew	*Crocidura nigeriae*	Least concern	*	Treatment of diseases believed to be caused by dirty spirits	Viruses: Hantaviruses, Langya henipavirus, Lassa virus, Borna disease virus 1; Bacteria: *Rickettsia* spp. [53,54]
African giant rat	*Cricetomys* spp.	Least concern	Head Whole	For the treatment of headache To cure barrenness in married women	Viruses: Lassa virus; Bacteria: *Leptospira* spp., *Bartonella* spp. [53,55]
Senegal Chameleon	*Chamaeleo senegalensis*	Least concern	*	For the treatment of pain	Bacteria: *Salmonella* spp. [56]
African Hare	*Lepus* spp.	Least concern	Fore and hindlimbs	For strengthening athletes’ bones For the treatment of osteoporosis	Bacteria: *Francisella tularensis*, *Yersinia pseudotuberculosis*; Parasites: *Toxoplasma gondii* [57]
Red-fronted Gazelle	*Eudorcas rufifrons*	Vulnerable	Hindlimbs	To improve athletes’ performance	Unknown
Nile monitor	*Varanus niloticus*	Least concern	Tail	For anti-poison	Bacteria: *Salmonella* spp., *Leptospira* spp.; Parasites: *Angiostrongylus cantonensis* [42,58]
Frugivorous bats	*Eidolon helvum*; *Hypsignathus monstrosus*; *Epomophorous gambianus*	Near threatened	Head; Wings; Whole	For the treatment of asthma, to enhance success and give one an advantage in business. To improve fertility in women	Viruses: Ebola virus, Marburg virus, Nipah virus, SARS-CoV-2, MERS-CoV, Rabies virus, Hantaviruses; Bacteria: Enteropathogenic *E. coli* (EPEC) [59,60]
Insectivorous bats	*Hipposideros* spp.; *Rhinolophus* spp.; *Nycteris* spp.	Least concern			
West African Dwarf crocodile	*Crocodylus suchus*	Vulnerable	Tail bones; Scales Head	For protection against enemies, to enhance libido, and for antitoxin	Viruses: West Nile virus; Bacteria: *E. coli*, *Salmonella* spp., *Chlamydia* spp., *Mycobacterium* spp., *Aeromonas* spp. [61,62]
West African slender-snouted crocodile	*Mecistops cataphractus*	Critically endangered			
House rat	*Rattus rattus*	Least concern	Head; Whole	For curing from demons, to treat infertility in women	Viruses: Lassa virus, Seoul hantavirus; Bacteria: *Yersinia pestis*, *Leptospira* spp., *Rickettsia* spp. Parasites: *Trypanosoma cruzi*, *Hymenolepis* spp. [53,63]
White-backed Vulture	*Gyps africanus*	Endangered	Head Feathers	For curing epilepsy, short-sightedness, mental ailments, and to give one good luck	Bacteria: *Clostridium perfringens*, *Mycobacterium* spp., *Campylobacter* spp., *Salmonella* spp., *Chlamydia psittaci* [64,65]
Hooded vulture	*Necrosyrtes monachus*	Critically endangered			
Brush-tailed Porcupine	*Atherurus africanus*	Least concern	Spikes	For spiritual protection	Bacteria: *Leptospira* spp.; Parasites: *Blastocystis* spp. [66,67]
African spurred tortoise	*Centrocelys sulcuta;*	Vulnerable	Whole	Treatment of ailments believed to be of spiritual origin	Bacteria: *Salmonella* spp.; Parasites: *Cryptosporidium* spp. [68,69]
Home’s hinge-back tortoise	*Kinixys homeana*	Critically endangered			
West African black mud turtle	*Pelusios niger*	Near threatened			
Tree hyrax	*Dendrohyrax* spp.	Least concern	Head	For spiritual protection	Bacteria: *Leptospira* spp. [53]

* Participant declined to answer.

## Data Availability

Data presented in this study are available on request from the corresponding author due to the cultural sensitivity and need to protect the privacy of the study subjects.

## Supplementary Materials

The following supporting information can be downloaded at: https://www.mdpi.com/article/10.3390/ani16030502/s1, File S1—Project information sheet (PIS), File S2—Questionnaire, File S3—Informed consent form (ICF), File S4—Focus group discussion (FGD) guide.
