# Supplementary material for: Use of Animal-Derived Products for Medicinal and Belief-Based Purposes in Urban Cities of Southwestern Nigeria: A One Health Perspective
Source: Animals (Basel). 2026 Feb 5;16(3):502. doi: 10.3390/ani16030502 (PMC12896710; doi:10.3390/ani16030502)
Supplement: Supplementary file 1 [file animals-16-00502-s001.zip › File S2.pdf]

Consent Form for Participation in a Research Study:
"Health risks associated with Urban Wildmeat in Lagos, Nigeria."

The investigator must explain this study to you before you agree to take part. If you have any questions arising from the information given to you, please ask the investigator conducting the research before you decide whether to take part or not. You will be given a copy of this form to keep and refer to at any time.

Documentation of Consent:

I have read or received information from this form and decided that I will participate in the research project described in this form. Its general purposes, the particulars of involvement and possible risks and inconveniences have been explained to my satisfaction. I understand that I do not have to participate in the study and can withdraw at any time. My signature also indicates that I have received a copy of this consent form. Each page of the consent form is initiated by me and the study staff, to indicate that the study staff has reviewed all of the pages with me.

By ticking each box, you are consenting to the elements of this study. It will be assumed that unticked box means that you DO NOT consent to that part of the study, and this may make you ineligible to participate in the study.

☐ I agree to allow the study staff initials on each page of the consent form.

There might be other research questions that investigators could study in the future with the help of samples like the one collected from the animals.

☐ I agree to fill the questionnaire, or take part in interview and discussions.

☐ I understand I will not be re-contacted about future potential use.

☐ I understand that my participation is voluntary and that I do not have to take part if I do not want to. I understand that I am free to withdraw from this study if I change my mind.

_____ _____
Subject name and signature Date

_____ _____
Parent/Legally Authorized Representative (if applicable) Date

I am unable to read but this consent document has been read and explained to me by _____ (name of reader). I therefore volunteer to participate in this research.

_____ _____
Subject name and signature Date

Consent Form for Participation in a Research Study:
"Health risks associated with Urban Wildmeat in Lagos, Nigeria."

_____ _____
Witness Date

Signature of Investigator or Responsible Individual:

To the best of my ability, I _____(name of investigator / person obtaining consent) have explained and discussed the full contents of the study, including all information contained in this consent form, and I have answered all questions from the research subjects and those of his/her parent(s) or legal guardian.

_____ _____
Signature Date
